# Supplementary material for: Identification of human transferrin receptor as an entry co-receptor for parvovirus B19 infection of human erythroid progenitor cells
Source: mBio. 2026 Jul 30;17(9):e01482-26. doi: 10.1128/mbio.01482-26 (PMC13556231; doi:10.1128/mbio.01482-26)
Supplement: Supplementary file 1 [file mbio.01482-26-s0001.pdf]

Supplemental Materials

A

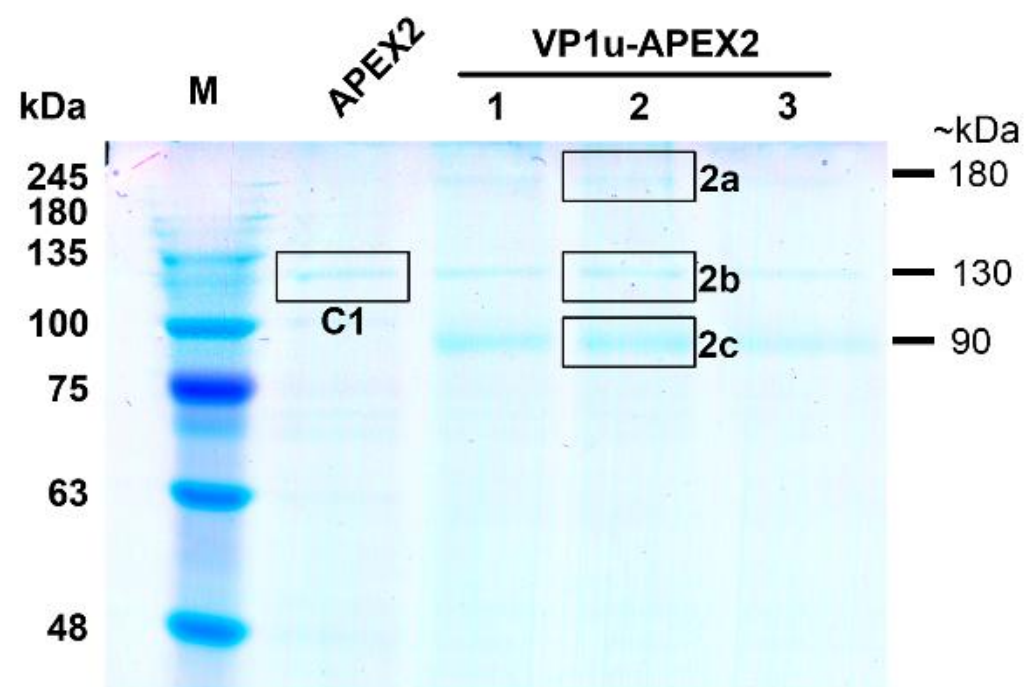

B

C1 (APEX2 control)				2a (VP1u-APEX2 unique band)				2b (Shared band)				2c (VP1u-APEX2 unique band)			
Unique reads	Total reads	Symbol	MW	Unique reads	Total reads	Symbol	MW	Unique reads	Total reads	Symbol	MW	Unique reads	Total reads	Symbol	MW
61	138	PC	129.55	44	71	TFRC	84.82	53	88	PC	129.55	63	174	TFRC	84.82
48	50	LRPPRC	157.81	42	42	ACACB	276.37	38	40	TFRC	84.82	30	30	PC	129.55
26	26	ACACA	265.38	30	30	PC	129.55	20	32	CDH1	97.4	25	38	CTNNB1	85.44
20	20	ACACB	276.37	24	27	NEO1	159.92	13	14	ITGB1	88.36	16	18	HSP90AA1	84.61
18	18	LARS1	134.38	23	24	FASN	273.03	12	13	ESYT1	122.78	12	12	CDH1	97.4
18	18	DHX9	140.87	12	12	ACACA	265.38	10	10	CTNNB1	85.44	10	10	HSP90AB1	83.21
14	14	ESYT1	122.78	11	11	IGF2R	274.2	10	10	ACTB	41.71	9	9	MCM4	96.5
14	14	HYOU1	111.27	11	11	FLNA	280.56	9	9	MYO1G	116.37	9	9	PDCD6IP	95.96
10	10	MCCC1	80.42	11	11	ANK1	206.14	8	8	MCM2	101.83	9	9	HSP90B1	92.41
9	9	MCM2	101.83	10	10	MCCC1	80.42	8	8	LRPPRC	157.81	8	8	MTHFD1	101.47

Figure S1. Identification of VP1u-APEX2 proximately biotinylated host proteins by SDS-PAGE and mass spectrometry.

A total of 1×10^7 UT7/Epo-S1 cells were incubated with 2 μM of VP1u-APEX2 or APEX2 control proteins at 37°C for 2 h. APEX2-mediated proximity biotinylation was then performed as described in the Materials and Methods. **(A) Coomassie blue staining biotinylated proteins.** Pulldown samples were resolved by SDS-PAGE and visualized by Coomassie blue staining to assess protein enrichment and to guide excision of bands (2a, 2b, and 2c) and a control band (0b), as indicated, for downstream mass spectrometry. **(B) Mass spectrometry identification of proteins from excised gel bands.** Tables list the top 10 proteins identified from each band based on unique peptide counts, with corresponding molecular weights (MW) indicated. TFRC (hTfR) was identified as the most enriched protein in Bands 2a and 2c, corresponding to monomer (~90 kDa) or dimer (~180 kDa) forms, respectively, and ranked second in band 2b (~130 kDa). The intermediate molecular weight of Band 2b likely reflects a differentially glycosylated form of hTfR. Additional proteins identified include cytoskeletal, chaperone, and membrane-associated factors.

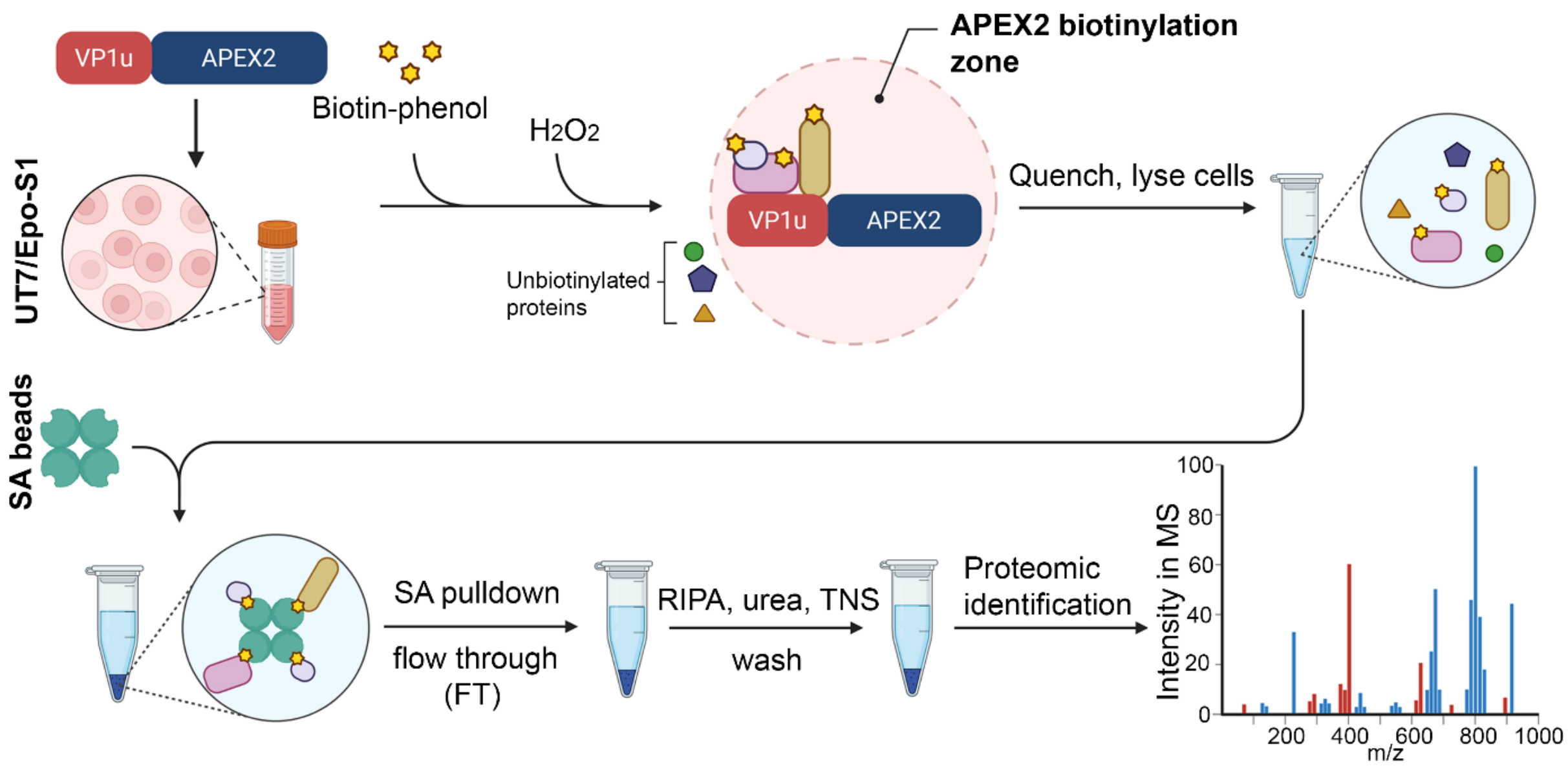

Figure S2. Diagram of workflow for APEX2-mediated proximity labelling.

UT7/Epo-S1 cells were incubated with VP1u-APEX2 or APEX2 control in the presence of biotin-phenol (at 37˚C for 30 min), followed by addition of H_2O_2 (1 min at room temperature) to induce biotinylation of proximal proteins. The reaction was then quenched, and the cells were lysed in RIPA buffer. The biotinylated proteins were enriched using streptavidin (SA) beads. After stringent washing (with RIPA, urea, and TNS buffers), bound proteins were subjected to on-bead enzyme digestion and proteomic identification by mass spectrometry.

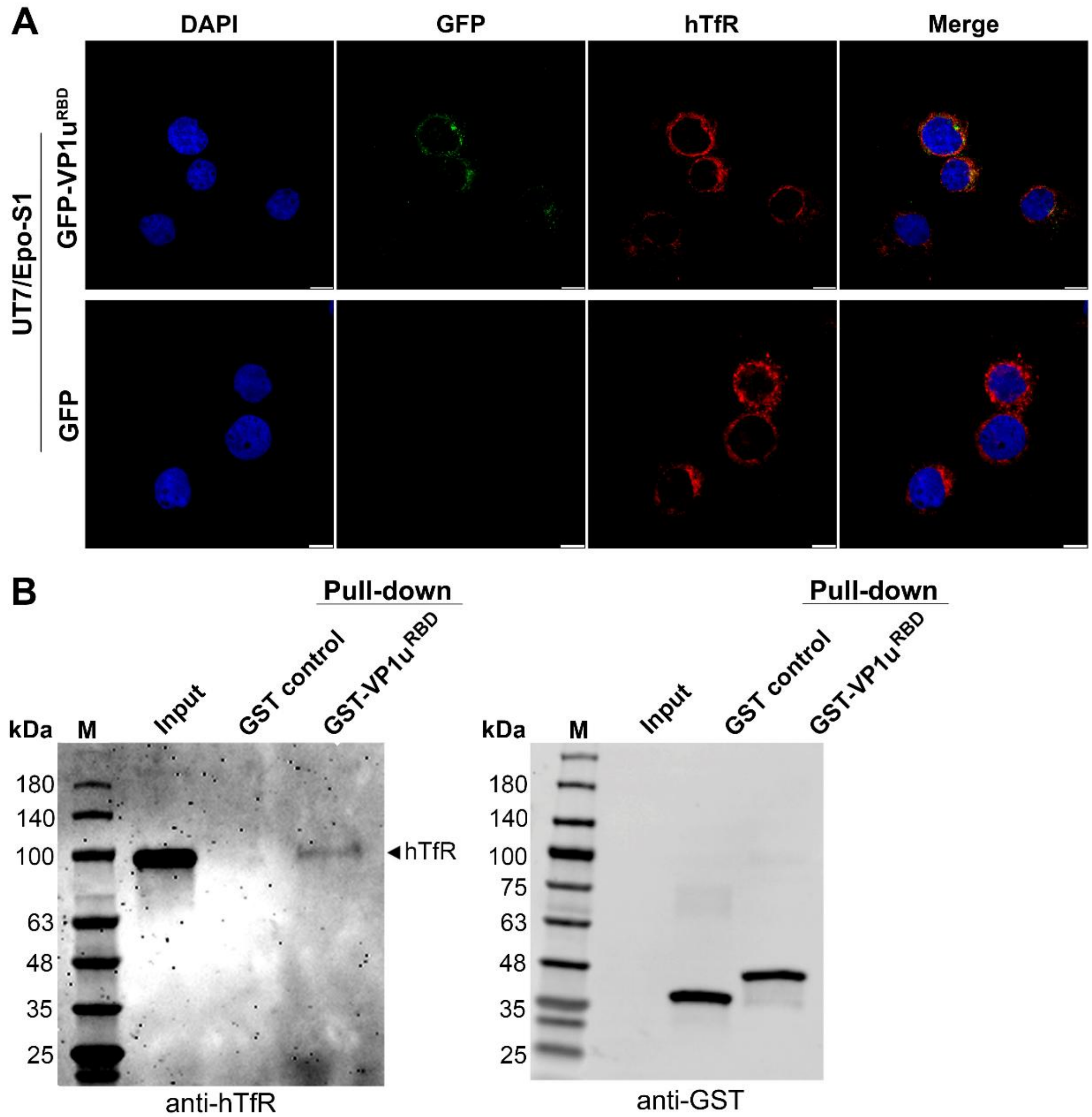

Figure S3. VP1u RBD colocalizes and interacts with hTfR in UT7/Epo-S1 cells.

(A) VP1u RBD colocalizes and interacts with hTfR. A total of 5 × 10^5 UT7/Epo-S1 cells were incubated with 1 μM purified GFP-$VP1u^{RBD}$ or GFP protein at 4˚C for 2 h with continuous rotation, followed by incubation at 37˚C for 10 min. The cells were then fixed, permeabilized, and stained with α-hTfR antibody. Nuclei were stained with DAPI. Bar = 10 μm. **(B) VP1u RBD interacts with hTfR.** GST-$VP1u^{RBD}$ or GST alone was immobilized on glutathione agarose beads in binding buffer (50 mM Tris-HCl, pH 8.0, 150 mM NaCl, 0.05% NP-40,1mM DTT) for 2 h at 4°C. The beads were washed > three times and incubated with UT7/Epo-S1 cell lysates for 8 h at 4°C. Bound proteins were eluted and analyzed by Western blotting. Left panel: Immunoblotting with an anti-hTfR1 antibody; right panel: Immunoblotting with an anti-GST antibody. Input represents UT7/Epo-S1 cell lysate prior to pull-down.

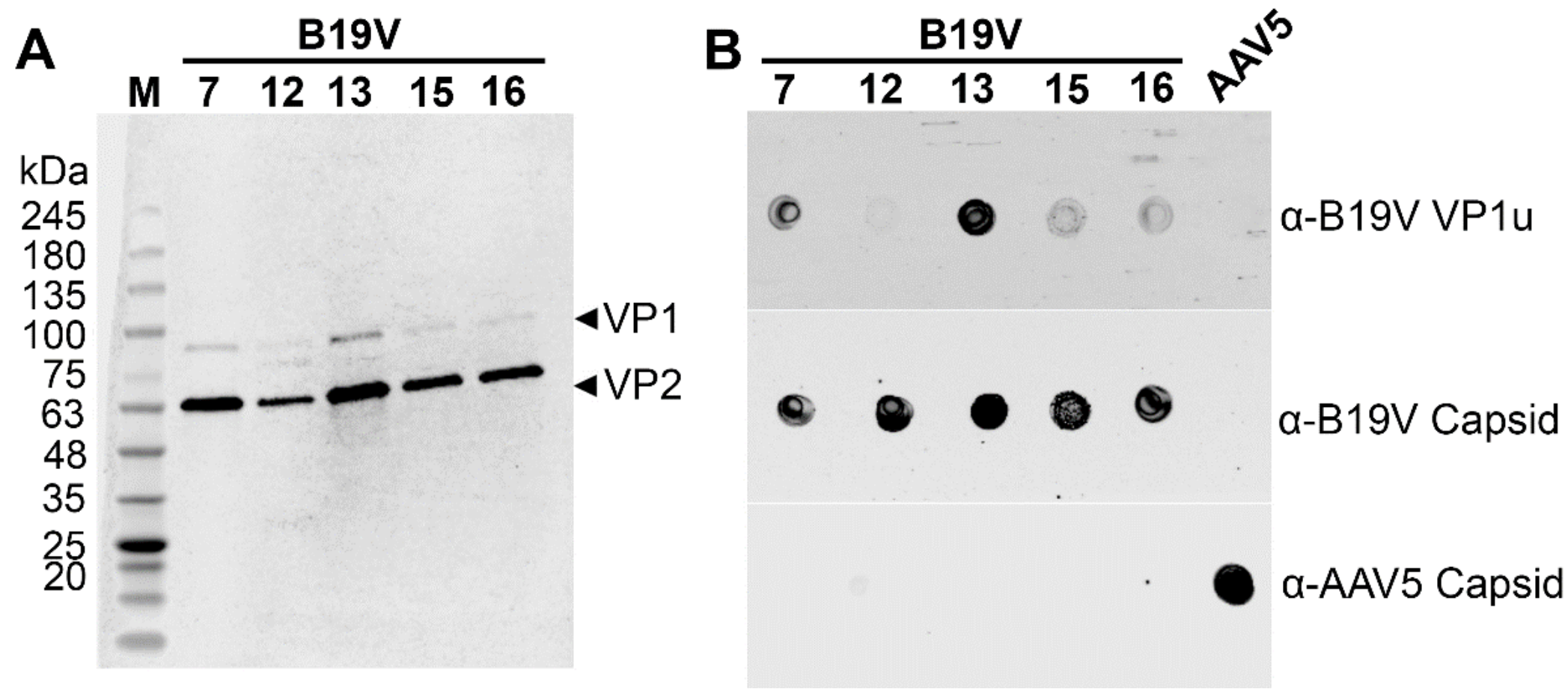

Figure S4. Characterization of purified B19V samples by Western blotting and dot blotting.

(A) Western blot analysis of purified B19V. B19V plasma samples (#7, 12, 13, 15, and 16) were purified in CsCl equilibrium ultracentrifugation. Approximately 1×10^9 viral genome copies (vgc) per sample were resolved by SDS–PAGE and probed with antibodies against B19V capsid proteins VP1 and VP2, as indicated. M, molecular weight marker (kDa). **(B) Dot blot analysis of B19V preparations.** Equal amounts (5×10^8 vgc) of each sample were spotted onto nitrocellulose membranes (Amersham) and probed with antibodies against B19V VP1u (α-B19V VP1u) and B19V capsid proteins (α-B19V capsid; Clone 521-5D). AAV5 was included as a specificity control and detected using an anti-AAV5 capsid antibody (Clone ADK5b).
